# Complexities in understanding attentional functioning among children with fetal alcohol spectrum disorder

**DOI:** 10.3389/fnhum.2014.00119

**Published:** 2014-03-07

**Authors:** Kimberly A. Lane, Jillian Stewart, Tania Fernandes, Natalie Russo, James T. Enns, Jacob A. Burack

**Affiliations:** ^1^Department of Educational and Counselling Psychology, McGill UniversityMontreal, QC, Canada; ^2^Department of Psychology, Syracuse UniversitySyracuse, NY, USA; ^3^Department of Psychology, University of British ColumbiaVancouver, BC, Canada

**Keywords:** fetal alcohol spectrum disorder, attentional control, selective attention, attention deficit hyperactivity disorder, test of everyday attention for children, prenatal exposure to alcohol, attention deficit, attention switching

## Abstract

Parental reports of attention problems and clinical symptomatology of ADHD among children with fetal alcohol syndrome disorder (FASD) were assessed in relation to performance on standardized subtests of attentional control/shifting and selective attention from the Test of Everyday Attention for Children (TEA-Ch; Manly etal., 1998). The participants included 14 children with FASD with a mean chronological age (CA) of 11.7 years and a mean mental age (MA) of 9.7 years, and 14 typically developing (TD) children with no reported history of prenatal exposure to alcohol or attention problems with a mean CA of 8.4 years and a mean MA of 9.6 years. The children with FASD were rated by their caregivers as having clinically significant attention difficulties for their developmental age. The reported symptomatology for the majority of the children with FASD were consistent with a diagnosis of ADHD, combined type, and only one child had a score within the average range. These reports are consistent with the finding that the children with FASD demonstrated difficulties with attentional control/shifting, but inconsistent with the finding that they outperformed the TD children on a test assessing selective attention. These findings are considered within the context of the complexity in understanding attentional functioning among children with FASD and discrepancies across sources of information and components of attention.

Common parental and anecdotal reports of general attentional problems among children with fetal alcohol spectrum disorder (FASD), a non-diagnostic umbrella term that refers to a spectrum of effects resulting from prenatal exposure to alcohol (PEA), seem consistent with high rates of clinical diagnoses of attention deficit hyperactivity disorder (ADHD) in this group. Yet, the complexity in evaluating general attentional processing from different perspectives and in relation to the many different components and theories of attention suggests a more nuanced reality. Accordingly, we examined the relationship among parental report of attentional problems and ADHD symptomatology in relation to performance on two standardized subtests of each of the essential attentional components of distractibility and cognitive control/switching among children with FASD as compared to typically developing (TD) children matched for mental age (MA).

The level of PEA is generally related to the extent of impairment associated with FASD, although the degree and type of impairment varies depending on a number of factors such as the timing of the PEA, maternal behaviors, and environmental conditions (Stratton et al., 1996; Chudley et al., 2005). Fetal alcohol syndrome (FAS), a specific pattern of birth defects associated with excessive maternal alcohol consumption during pregnancy, represents the most severe consequence of PEA. These birth defects, which historically included growth deficiency, a pattern of facial anomalies, and central nervous system dysfunction, were first described in the medical literature in the early 1970s (Jones and Smith, 1973; Jones et al., 1973). The criteria for FAS have remained largely the same during the past four decades, although they are now more clearly defined through the development of diagnostic procedures (e.g., Astley, 2004; Chudley et al., 2005; Hoyme et al., 2005).

According to the prevailing Canadian guidelines (Chudley et al., 2005), the term FASD includes the diagnoses of FAS, partial fetal alcohol syndrome (pFAS), and alcohol-related neurodevelopmental disorder (ARND). The diagnostic criteria for all three include PEA and significant brain dysfunction. A diagnosis of FAS also requires growth deficiency (i.e., weight and/or height <10th percentile) and certain characteristic facial features (i.e., short palpebral fissures, flat philtrum, and thin upper lip) along with PEA and significant brain dysfunction. Partial FAS is diagnosed when only two of the three characteristic facial features are present with or without growth deficiency. A diagnosis of ARND is provided when significant brain dysfunction has occurred as a result of PEA. Within this diagnostic framework, confirmed maternal alcohol use during pregnancy is necessary but not sufficient for an alcohol-related diagnosis, as brain dysfunction must also be evident. All individuals diagnosed with an alcohol-related disorder based on the Canadian guidelines are impacted by PEA and considered to have static encephalopathy (i.e., non-progressive brain damage) as a result.

Although all children with FASD present with broad deficits (i.e., significant impairment in three or more domains of brain functioning), a specific profile of brain dysfunction unique to FASD has not been identified (Chudley et al., 2005). Rather, the literature seems to point to a “generalized deficit in processing complex information” (Kodituwakku, 2007, p. 199; for examples, see Aragón et al., 2008), as evidenced by findings of a wide range of reported cognitive deficits associated with PEA, including those of general cognition (Coles et al., 1991), learning and language (Mattson and Riley, 1998), executive function (Rasmussen, 2005), visual-spatial processing (Olsen et al., 1998), memory (Coles et al., 2010), and attention (Lee et al., 2004). Similarly, in a 25 year longitudinal study on the effects of PEA in a primarily middle-class population in Seattle, Streissguth (2007) identified problems throughout childhood in attention, visual-spatial memory, speed of information processing, IQ level, and arithmetic. In particular, attentional difficulties have been cited as sequelae of prenatal alcohol exposure that lead to many of the concomitant difficulties that are reported among individuals with PEA (Kopera-Frye et al., 1997).

## THE ATTENTIONAL FUNCTIONING OF CHILDREN WITH PEA

The extent of the attention problems among children with FASD (e.g., Nanson and Hiscock, 1990; Oesterheld and Wilson, 1997; Malbin, 2002) led to a notion of attention problems as core deficits (Kopera-Frye et al., 1997). Disruption in attentional functioning as a consequence of PEA appears to be evident almost from birth (Streissguth, 2007); for example, PEA was significantly related to poor habituation to light in exposed human infants one or two days after birth (Streissguth et al., 1983). These problems appear to persist through infancy. In a study of the RTs (response times) of 6.5 month old infants drawn from a larger longitudinal study of the effects of PEA on infant cognition, Jacobson et al. (1994) found that prenatal alcohol exposure was associated with an increased latency to shift eye gaze to a visual stimuli after the stimulus was presented, which was thought to reflect slowed information processing. Similarly, in a study of attentional regulation among 6 month old infants with varying levels of PEA using cardiac-orienting responses in response to the presentation of auditory (tones) and visual (faces) stimuli, Kable and Coles (2004) found that infants whose mothers scored high on a substance abuse checklist took longer to reach the heart rate deceleration criteria following the onset of a new event as compared to infants whose mothers scored low on the checklist. This finding was thought to reflect difficulties in the initiation of attention, and suggested a decrease in the speed with which information is encoded.

Attention difficulties arising from PEA continue into childhood (e.g., Lee et al., 2004; Kooistra et al., 2010). For example, children with PEA often meet criteria for ADHD based on clinical interviews (Koren et al., 2003; Fryer et al., 2007; Kooistra et al., 2010), score higher than same-aged peers on behavioral questionnaires that assess attention problems (Nanson and Hiscock, 1990; Brown et al., 1991; Coles et al., 1997; Lee et al., 2004; Nash et al., 2006; Astley et al., 2009), and are rated as more inattentive at school than children of mothers who did not (Brown et al., 1991). Yet, even as children with PEA consistently present with behavioural symptoms of inattention (e.g., Fryer et al., 2007), they do not always demonstrate deficits on experimental or clinical measures of attentional functioning. One reason for this discrepancy might be that children with PEA who exhibit externalizing problems and hyperactivity are more difficult to manage and are, therefore, more likely to be referred to a clinic for diagnosis and treatment (Coles et al., 1997).

### Attention control/shifting attention

Mirsky et al. (1991) defined the shift component of attention as the “ability to change attentive focus in a flexible and adaptive manner” (p. 112), and performance on the Wisconsin Card Sorting Task (WCST) was used to measure this aspect of attention in their model. Performance on the WCST appears to be related to rates of PEA with deficits commonly found among those with greater (Kodituwakku et al., 1995, 2001b; Coles et al., 1997; McGee et al., 2008; Vaurio et al., 2008), but not lower (Richardson et al., 2002; Burden et al., 2005), levels of PEA. For example, Astley et al. (2009) found that children with FASD who would be considered to have an alcohol-related disorder according to the Canadian diagnostic guidelines made significantly more errors on a computerized version of the WCST than both children without PEA and children with *mild ARND *(i.e., defined as PEA but significant impairment in less than three areas of brain function). Similarly, Connor et al. (2000) found that a clinical group of diagnosed adults with PEA consistently demonstrated extreme deficits on the WCST, whereas adults with lower levels of PEA did not.

The WCST may also not be a good measure of attention shifting for children with FASD, as it is a complex task that relies on broader abilities than attention, especially executive function which appears to be an area that is impaired among individuals with PEA (Connor et al., 2000; Kodituwakku et al., 2001a; Rasmussen, 2005). Accordingly, in an attempt to use an alternative paradigm of attention shifting, Mattson et al. (2006) administered a computerized experimental task that involved both the visual and auditory modalities to 9–14 years old children considered to have experienced heavy PEA. The children exposed to high levels of prenatal alcohol were slower than the TD children when required to switch back and forth between the auditory (high tone, low tone) and visual (red square, green square) stimuli that were each presented one at a time with varying interstimulus time intervals. As they were not less accurate than the TD children when full scale IQ was used as a covariate, Mattson et al. (2006) suggested that children with FASD were capable of switching between modalities, but that it required more cognitive effort for them.

Difficulties in shifting attention are supported by the performance of children with heavy PEA on other measures that involve an aspect of switching. For example, Vaurio et al. (2008) found that children with PEA who also met criteria for ADHD demonstrated significant difficulties in comparison to both TD children and children with ADHD on the Trail Making Test – Part B (e.g., Reitan and Wolfson, 1993) which requires switching between sequencing a set of numbers and letters. These findings are consistent with the performance of children diagnosed with an alcohol-related disorder. For example, Rasmussen and Bisanz (2009) and Astley et al. (2009) found that the children with FASD demonstrated significant difficulties switching between letters and numbers on the Trail Making Test from the Delis-Kaplan Executive Function System (Delis et al., 2001).

### Selective attention

Selective attention refers to the ability to direct attentional resources to a task and filter distracting stimuli (Mirsky et al., 1991). Children (Burden et al., 2005), adolescents (Streissguth et al., 1994), and adults (Connor et al., 1999) with PEA demonstrate difficulties on digit cancellation tasks used to assess selective attention. The attention shifting task administered by Mattson et al. (2006) also included visual and auditory focused attention conditions that required the participants to maintain focused attention to stimuli in one modality while ignoring visual and auditory distracters. Mattson et al. (2006) found that the children with PEA were less accurate in the focused attention conditions and consistently responded slower to visual stimuli than TD children, indicating a “consistent and significant deficit in visual focused attention” (p. 366).

## THE PRESENT STUDY: ATTENTIONAL CONTROL AND SELECTIVE ATTENTION AMONG CHILDREN WITH FASD

The aim of the present study was to examine the attentional functioning of children with FASD in relation to MA matched TD children. We used a strict criterion for measuring PEA, by including only those children diagnosed with an alcohol-related disorder, rather than children exposed to prenatal alcohol. Children diagnosed with an alcohol-related disorder using the Canadian guidelines (Chudley et al., 2005) have been exposed to prenatal alcohol and are also *affected* by the exposure. This distinction is particularly important in the search for deficits exhibited by children with FASD, since not all children exposed to prenatal alcohol are later identified with FASD (Stratton et al., 1996). The dosage and timing of the prenatal alcohol experienced by children in this study, although not measured specifically, was sufficient to produce brain dysfunction.

The issue of developmental level was addressed by comparing the performance of children with FASD with the performance of TD children at the same developmental level, as indicated by MA as measured by the Leiter International Performance Scale – Revised (Leiter-R; Roid and Miller, 1997). Due to the typically lower MAs among children with FASD, comparing them with TD children of the same chronological age (CA) is potentially misleading, particularly on abilities such as those of visual attention in which developmental changes occur (e.g., Enns and Girgus, 1985; Pastò and Burack, 1997). Thus, comparisons with TD children of the same MA allow researchers to determine whether attentional performance is developmentally appropriate or problematic in relation to a priori differences in level of functioning that are not linked to FASD per se (for a discussion of relevant issues, please see Burack et al., 2004). In this study, the Leiter-R (Roid and Miller, 1997), an entirely non-verbal visual measure of cognitive ability, was used to estimate developmental level. Using this measure, children with FASD were matched to TD children on MA (mental age) so that group differences could then be attributed to characteristics unique to the children with FASD. 

Attention is one of the brain domains recommended to be assessed during the neuropsychological assessment for FASD (Chudley et al., 2005); significant impairment in this domain could reflect a clinical diagnosis of ADHD and/or poor performance on clinical measures that require attention. In our study, the Conners’ Rating Scale (Conners, 1997) was used to assess behavioral symptoms of ADHD, and subtests from the Test of Everyday Attention for Children (TEA-Ch; Manly et al., 1998) were used to assess visual attention, particularly selective attention, and attentional control/shifting. The TEA-Ch was considered an appropriate choice for children with FASD, as the test was designed to measure various components of attention without relying on other abilities, such as memory, verbal comprehension, or motor speed (Manly et al., 2001), any of which might be impaired in children with FASD (e.g., Stratton et al., 1996). 

## MATERIALS AND METHOD

### PARTICIPANTS

The participants included 14 children (9 females) with FASD with a mean CA of 11.73 (SD = 1.36) years, a mean MA of 9.65 (1.47) years, and a mean brief non-verbal IQ (intelligence quotient) of 83 (10.59), and 14 TD children (9 females) with no reported history of PEA or attention problems with a mean CA of 8.42 (1.39) years, a mean MA of 9.59 (1.55) years, and a mean brief non-verbal IQ of 114.93 (9.92). The groups were matched on gender and within four months of MA based on the Leiter-R (Roid and Miller, 1997), a standardized measure of non-verbal intelligence. The children with FASD did not differ from the TD children on mean MA, *t*(26) = 0.115, *p* = 0.909, but were significantly older, *t*(26) = 6.364, *p* = < 0.001, and had significantly lower non-verbal IQs, *t*(26) = -8.217, *p* = < 0.001. Descriptive statistics for the two groups are presented in **Table 1**.**

**Table 1:** Descriptive Statistics for the participants with FASD and TD participants.

Group	*N*	CA		MA		Brief non-verbal IQ		% Male	% Caucasian
		*M*	*SD*	*M*	*SD*	*M*	*SD*
FASD	14	11.73	1.36	9.65	1.47	83.07	10.59	35.7	57.1
TD	14	8.42	1.39	9.59	1.55	114.93	9.92	35.7	92.9

*CA, chronological age; MA, mental age; Brief IQ, brief non-verbal IQ score from the Leiter-R.
*

The children with FASD were recruited from the Asante Centre for Fetal Alcohol Syndrome, a FASD assessment and diagnostic centre located in the Fraser Region of British Columbia (BC) that provides assessment to individuals throughout BC. A staff member from the Asante Centre contacted legal guardians of children between 8 and 13 years of age who underwent a FASD assessment through the centre, and invited them to participate in the study. Twenty-two children were initially tested, but eight were eliminated from the study as the MAs of five children fell outside of the target developmental age range for this study (i.e., 7 to 12 years), two children did not have confirmed PEA, and a TD match was not found for one child. All of the children with FASD had been assessed in accordance with the Canadian diagnostic guidelines (Chudley et al., 2005) and received one of three alcohol-related diagnoses, FAS (*n* = 1), pFAS (*n* = 3), or ARND (*n* = 10). Eight of the participants with FASD were rated by the diagnostic team as having significant attention problems, four were rated as having mild to moderate attention problems, and only one was rated as having no attention problems (data for one participant was missing). Nine of the children with FASD had a confirmed diagnosis of ADHD. None of the children with FASD were living with their mothers and only two with their birth fathers; 6 with foster families; 4 with adoptive families; two with relatives. All of the children for whom the information was available (*n* = 12) experienced postnatal risk (e.g., multiple placements; abuse/neglect). Ten of the children for whom the information was known (*n *= 11) experienced other prenatal exposures in addition to alcohol (e.g., tobacco; marijuana). Five children regularly took medication to manage their attentional difficulties and the caregivers of these children were asked to not give the medication on the day of testing. Three of these children were tested off their medication. Two were on medication during the time of the assessment (one because the caregiver forgot and one because of the type of medication). The children who were tested off their medication had taken their last dose at least 24 hours before the testing session.

The TD children were recruited from communities in British Columbia through the use of community postings, school contacts, and the distribution of flyers to acquaintances and colleagues. Only a parent or caregiver knowledgeable about the child’s prenatal history were included in the study.

### MEASURES

#### The Leiter International Performance Scale – Revised (Leiter-R)**

The Leiter-R (Roid and Miller, 1997) is a non-verbal measure of cognitive ability developed for use with individuals from 2 to 20 years of age. The Leiter-R is entirely non-verbal and performance is not timed. It is comprised of 20 subtests organized into the two major areas of Reasoning and Visualization (10 subtests), and Attention and Memory (10 subtests). Standard scores are generated for each of the composites under these major areas. The Brief IQ Composite (four subtests) was used to estimate the developmental level or the MA of the participants in this study.

#### The Conners’ Rating Scale: Long Version – Parent Form (CPRS:L)

The CPRS:L (Conners, 1997) is a rating scale administered to caregivers of children and adolescents to aid in the assessment of ADHD and other comorbid issues. The CPRS:L includes scales that correspond to the DSM-IV diagnostic criteria for ADHD. The results of this rating scale were used as a measure of the degree to which each child displays clinically significant attention problems.

#### The Test of Everyday Attention for Children (TEA-Ch)

The TEA-Ch (Manly et al., 1998) was designed to assess various components of attention in children. The TEA-Ch is comprised of nine subtests that are used to measure focused (selective) attention, sustained attention, and attentional control/switching. The tasks are “game-like” and require little memory or verbal comprehension skills, which makes the TEA-Ch a potentially appropriate tool for use with children with disabilities such as FASD. The four subtests that involve visual attention were administered in this study. Two of the subtests involved selective attention (Sky Search and Map Mission), and the other two involved attentional control/switching (Creature Counting and Opposite Worlds). Norms from the TEA-Ch which were derived from 293 children and adolescents between the ages of 6 and 16 years.

(1) On the *Sky Search* subtest, the children were required to quickly circle target pairs among distracters on paper. Sky Search includes a trial with no distracters in order to control for motor speed. Scores on the Sky Search task are based on the number of correctly identified targets, as well as the amount of time taken to identify each target.
(2) On the *Map Mission *subtest, the children were required to locate as many target stimuli as possible on a city map within a time limit. Map Mission scores are based on the number of target shapes correctly identified on a display.
(3) On the *Creature Counting *subtest, the children were required to switch between counting forward and backward in response to visual targets. Participant scores are based on accuracy in counting, and time taken to complete the task.
(4) ) On the *Opposite Worlds* subtest, the children were first required to name aloud the numbers “1” and “2” that they saw displayed along a path on paper. In the “opposite world” they were required to say “1” when they saw a “2,” and say “2” when they saw a “1.” The scores on this subtest are based on the time taken to correctly complete the task.

### PROCEDURE 

Ethics approval was obtained from the Human Research Ethics Board at McGill University. The legal guardians and caregivers (when different) provided signed informed consent prior to testing. Verbal assent was also obtained from the participating child. In the case of the TD participants, the child’s parent completed a brief questionnaire to confirm that the child did not experience prenatal substance exposure, or have a history of learning, behavior, or attentional problems. The alcohol-related diagnosis, ratings for the attention-deficit hyperactivity brain domain and the postnatal risk, and other prenatal substance exposures for each of the children with FASD were obtained from the Asante Centre diagnostic assessment file. 

All of the children were tested in a quiet room with limited distractions. The majority of the children with FASD were tested at the Asante Centre. One participant was tested in their home and another participant was tested at another community agency. The TD children were either tested at the Asante Centre, another community agency, or their school. All of the assessment measures were administered by an experienced clinician trained in test administration. 

## RESULTS

### CAREGIVER RATINGS OF ATTENTION DIFFICULTIES

The mean ratings on the Conners’ scale for each group are presented in **Table 2**. As expected, the participants with FASD were rated by their caregivers on the Conners’ as having clinically significant attention difficulties for their developmental age. This was not the case for the MA-matched TD participants, who had significantly lower *T*-scores on all subscales. These conclusions were supported by the following evidence. The caregiver reported significant cognitive problems/inattention in relation to their MA (*M *= 79.08, *SD* = 8.78; range 67–90) for all of the children with FASD. None of the children with FASD scored within the average range on the diagnostic-oriented scale for ADHD, inattentive type, and only two scored within the average range on the diagnostically oriented scale for ADHD, hyperactive-impulsive type. The reported symptomatology for the majority (*n* = 10; 76.9%) of the children with FASD (*n* = 13) were consistent with a diagnosis of ADHD, combined type, as measured by the Conners’ (i.e., *T*-score of 70 or above), and only one child had a score within the average range. None of the TD children displayed symptoms of ADHD. 

**Table 2:** Mean *T*-scores (standard deviations) based on MA for both groups on the Conners’ subscales.

	FASD (*n* = 13)		TD (*n* = 12)	
Conners’ subscale	*M*	*SD*	*M*	*SD*
Cognitive problems/inattention	79.08	8.78	45.75	2.22
DSM-IV index: inattentive	77.00	9.97	45.50	2.88
DSM-IV index: hyperactive-impulsive	72.92	13.20	50.92	4.34
DSM-IV index: total	76.77	10.66	47.75	3.31

### GROUP COMPARISON ON THE TEA-Ch

The mean scores for each of the subtests from the TEA-Ch, which were administered to assess visual selective attention (the Sky Search and Map Mission subtests) and attention control/switching (the Opposite Worlds and Creature Counting subtests) are presented in **Table 3**. Differences between the groups as assessed by *t*-tests were found for only two comparisons. Inconsistent both with previous evidence from children with FASD and with their behavioral presentation, the children with FASD as a group scored within the average range for their developmental level on all but one of the standardized subtests (Creature Counting). The finding of average levels of focused attention on the TEA-Ch subtests in relation to developmental level is consistent with evidence from children with ADHD (Heaton et al., 2001).

**Table 3:** Comparison of mean TEA-Ch subtest scores (calculated based on MA) between FASD and TD groups.

	FASD		TD	
Subtest	*n*	*M*(SD)	*n*	*M*(SD)	*t*	*p*
**Selective attention subtests**
Sky search						
Correct	14	10.36 (2.21)	14	8.86 (2.35)	1.742	0.093
Timing per correct target	14	9.21 (2.94)	14	7.71 (2.34)	1.495	0.147
Map mission						
Targets found	14	11.79 (3.09)	14	8.86 (3.06)	2.519	0.018
**Attention control/switching subtests**
Creature counting						
Correct	14	5.64 (1.65)	13	9.62 (3.82)	-3.463^a^	0.003
Timing	7	10.14 (3.08)	12	9.67 (3.80)	0.281	0.782
Opposite Worlds						
Same World	14	9.14 (2.57)	13	9.46 (3.93)	-0.251	0.804
Opposite World	14	8.79 (3.22)	13	8.31 (3.52)	0.369	0.715

a df = 16.05 (unequal variances).

#### Group comparisons on measures of attention control/shifting

The children with FASD and TD children did not differ in speed or accuracy on the Opposite Worlds task [Same World *t*(25) = -0.251, *p* > 0.05; Opposite World *t*(25) = 0.469, *p* > 0.05], however, the children with FASD demonstrated difficulties on Creature Counting as only three children with FASD performed within the average range for their MA on the accuracy component of this subtest. The children with FASD were less accurate in their counting than TD children matched on MA [*t*(16.05) = -3.463, *p* = 0.003], but did not differ from TD children on time taken to complete the task [*t*(17) = 0.281, *p* > 0.05]. However, timing was counted only for the seven children with FASD who accurately answered more than two of the seven trials.

#### Group comparisons on measures of selective attention

The children with FASD outperformed the TD children on the Map Mission task [*t*(26) = 2.519, *p *= 0.018], but did not differ from them on the time taken to find each target [*t*(26) = 1.495 *p* > 0.05] on the Sky Search task. The children with FASD also outperformed the TD children on the number of targets found on the Sky Search task, but this difference did not reach the conventional levels of statistical significance [*t*(26) = 1.742, *p* = 0.09].

## Discussion

The findings from this study provide insight into the complexity of the real-world perceptions and manifestations of attentional processing among children with FASD. This complexity is manifest as a gap between everyday observational and clinical methods of assessment. Consistent with previous evidence, the 14 children with FASD in this study, all of whom were impacted by PEA as assessed with the Canadian diagnostic guidelines (Chudley et al., 2005) and functioned at MAs between 7 and 12 years, received high ratings of attention problems by their caregivers that were commensurate with a high incidence of a clinical diagnosis of ADHD. Yet, their performance on clinical subtests of attention from the TEA-Ch (Manly et al., 1998) reflected a more nuanced pattern of attentional functioning. 

These results also highlight the need to provide more fine-tuned accounts that include multiple sources of information about various components of attention. Consistent with previous evidence that individuals with FASD, especially those with high rates of PEA, appear to have difficulties with attentional shifting (e.g., Coles et al., 1997; Kerns et al., 1997; Kodituwakku et al., 2001a, b; Mattson et al., 2006), the children with FASD in this study performed below average for their MA, and significantly worse than the MA-matched TD children, on the Creature Counting subtest of the TEA-Ch, which is used to assess task switching, in this case, between counting forward and counting backward. However, this diminished performance might also be a function of the difficulties associated with arithmetic that have been reported among children with FASD (Streissguth et al., 1994; Goldschmidt et al., 1996; Howell et al., 2006; Jacobson et al., 2011).

In contrast, based on their performance on the Sky Search and Map Mission subtests of the TEA-Ch, the children with FASD demonstrated an ability to attend to relevant stimuli in the presence of distracters at a level that appeared to be consistent with their MA as based on non-verbal cognitive ability. Although discrepant with findings of impaired selective attention among children with FASD (Connor et al., 1999; Streissguth et al., 1999; Burden et al., 2005; Mattson et al., 2006), the findings of average or better levels of performance reported here are consistent with evidence that children with ADHD also perform within the average range on the visual selective attention subtests of the TEA-Ch (Heaton et al., 2001; Manly et al., 2001). As the behavior of both children with ADHD and those with FASD is characterized as distractible and inattentive (American Psychiatric Association, 1994; Hudziak et al., 2004), the commensurate findings from the two groups suggest that the subtests of the TEA-Ch may be measures of selective attention that are not confounded with other aspects of attention, such as vigilance or control, that have been cited as the source of the attentional problems at least among children with ADHD (Manly et al., 2001). Conversely, the TEA-Ch subtests may not be sufficiently sensitive to detect nuanced real-world attentional problems. Additionally, differences in methodologies, such as matching on CA rather than MA (Connor et al., 1999) and using RT, rather than accuracy, to assess selective attention performance (Mattson et al., 2006) could account for the discrepancies between this and other studies with regard to performance on the TEA-Ch by children with FASD.

The implications of this study must be considered within the constraints of research on persons with FASD. Due to the difficulties in recruiting participants who met the guidelines for FASD and were able to complete the task, the number of participants in this study precluded comparisons among subgroups with regard to variables such as the specific FASD diagnosis, gender, diagnosis of ADHD, medication history, developmental level, living situation, and other life circumstances. The findings may also have been affected by maternal smoking during pregnancy which was not considered in this study but has possible links with ADHD symptoms in children (Thapar et al., 2003; Langley et al., 2005), and, therefore, may account for some of the observed attentional difficulties among the children with FASD in this study. As is common, the group of children with FASD had a mean IQ score in the low average range, and therefore, was matched to the group of TD children on MA in order to ensure that any of the expected deficits in attention would be specific to the task rather than to a priori differences in developmental level. Although the inevitable outcome is that the children with FASD were chronologically older, this type of MA matching is advocated among developmental researchers in the study of attention and related areas of functioning among persons with lower IQ levels (for reviews, see Iarocci and Burack, 1998; Burack et al., 2001, 2004, 2013). The shortcoming of this matching strategy is that it eliminates the possibility of controlling for differences in verbal proficiency between participants, although the impact of verbal differences on our findings was likely minimal as the tasks were non-verbal and were successfully completed by the participants. In addition, despite being a common methodological practice, our a priori exclusion of TD children with documented attention problems may have exacerbated the finding of any group differences in attentional functioning between the groups. 

In sum, these findings highlight three points essential to understanding the development of attention among children with FASD. One, the level of functioning exhibited by a child with FASD varies considerably, depending on which component of attention is assessed. Two, the clinical assessment of attentional problems as they are expressed in everyday life may be misleading when they are made in comparison to peers of the same CA, rather than the more appropriate comparison to peers of the same MA, which is a more accurate reflection of level of functioning for children with FASD whose general cognitive level is often lower than that of their peers. Thus, the CA comparisons would lead to both everyday impressions and clinical diagnoses of hyperactivity and ADHD, although the children might be behaving more appropriately in relation to MA. Three, parent, clinical, and experimental information are often quite discrepant, partly because they each tap into different aspects of functioning, and partly because they entail different premises of inference. 

## Conflict of Interest Statement

The authors declare that the research was conducted in the absence of any commercial or financial relationships that could be construed as a potential conflict of interest.
